# Dutch Young Adults Ratings of Behavior Change Techniques Applied in Mobile Phone Apps to Promote Physical Activity: A Cross-Sectional Survey

**DOI:** 10.2196/mhealth.4383

**Published:** 2015-11-12

**Authors:** Laura S Belmon, Anouk Middelweerd, Saskia J te Velde, Johannes Brug

**Affiliations:** ^1^ EMGO Institute for Health and Care Research Department of Epidemiology and Biostatistics VU Medical Center Amsterdam Amsterdam Netherlands

**Keywords:** motor activity, self efficacy, exercise, behavior therapy, cell phones

## Abstract

**Background:**

Interventions delivered through new device technology, including mobile phone apps, appear to be an effective method to reach young adults. Previous research indicates that self-efficacy and social support for physical activity and self-regulation behavior change techniques (BCT), such as goal setting, feedback, and self-monitoring, are important for promoting physical activity; however, little is known about evaluations by the target population of BCTs applied to physical activity apps and whether these preferences are associated with individual personality characteristics.

**Objective:**

This study aimed to explore young adults’ opinions regarding BCTs (including self-regulation techniques) applied in mobile phone physical activity apps, and to examine associations between personality characteristics and ratings of BCTs applied in physical activity apps.

**Methods:**

We conducted a cross-sectional online survey among healthy 18 to 30-year-old adults (N=179). Data on participants’ gender, age, height, weight, current education level, living situation, mobile phone use, personality traits, exercise self-efficacy, exercise self-identity, total physical activity level, and whether participants met Dutch physical activity guidelines were collected. Items for rating BCTs applied in physical activity apps were selected from a hierarchical taxonomy for BCTs, and were clustered into three BCT categories according to factor analysis: “goal setting and goal reviewing,” “feedback and self-monitoring,” and “social support and social comparison.”

**Results:**

Most participants were female (n=146), highly educated (n=169), physically active, and had high levels of self-efficacy. In general, we observed high ratings of BCTs aimed to increase “goal setting and goal reviewing” and “feedback and self-monitoring,” but not for BCTs addressing “social support and social comparison.” Only 3 (out of 16 tested) significant associations between personality characteristics and BCTs were observed: “agreeableness” was related to more positive ratings of BCTs addressing “goal setting and goal reviewing” (OR 1.61, 95% CI 1.06-2.41), “neuroticism” was related to BCTs addressing “feedback and self-monitoring” (OR 0.76, 95% CI 0.58-1.00), and “exercise self-efficacy” was related to a high rating of BCTs addressing “feedback and self-monitoring” (OR 1.06, 95% CI 1.02-1.11). No associations were observed between personality characteristics (ie, personality, exercise self-efficacy, exercise self-identity) and participants’ ratings of BCTs addressing “social support and social comparison.”

**Conclusions:**

Young Dutch physically active adults rate self-regulation techniques as most positive and techniques addressing social support as less positive among mobile phone apps that aim to promote physical activity. Such ratings of BCTs differ according to personality traits and exercise self-efficacy. Future research should focus on which behavior change techniques in app-based interventions are most effective to increase physical activity.

##  Introduction

Despite its well-known benefits, 31% of adults worldwide and 40% of Dutch adults do not engage in sufficient physical activity [1-3]. In early adulthood (18-30 years of age), levels of physical activity often decline from childhood levels [4-6]. Therefore, widely available, effective, and affordable public health interventions are needed to promote physical activity in this age group.

In 2013, 59% of Dutch young adults owned a mobile phone [7]. As ownership and utilization of mobile phones increase, more people are accepting the use of mobile health apps and the popularity of physical activity apps is increasing [8,9]. In January 2015, the iTunes and Google Play stores contained 40,868 and 43,092 health and fitness apps, respectively [10,11]; such apps may be useful for promoting physical activity.

There is, however, limited evidence on the effectiveness of app-based interventions; previous research shows that Web-based interventions grounded in behavior change theory are more likely to be effective [12], but most presently available apps aiming to promote physical activity are not theory-based and do not address the most important behavioral determinants [9,13,14]. Previous research also indicates that self-efficacy and social support are both associated with physical activity and should be addressed when aiming to increase physical activity behavior [15]. Furthermore, previous research suggests that individually tailored health messages may be more effective than nontailored generic messages [16-19].

Behavior change techniques (BCTs) are systematic procedures included as an active component of an intervention designed to change behavior [20,21]. BCTs aim to address behavioral determinants such as self-efficacy. Michie et al [22] developed the Behavior Change Technique Taxonomy (v1) of 93 Hierarchically Clustered Techniques; they reported that self-regulation techniques (eg, self-monitoring, action planning, providing instruction, reinforcing effort toward behavior, goal setting, goal reviewing, and providing feedback on behavior) and planning social support or social change [23-26] appear to be mainly important to increase self-efficacy and social support for physical activity in individual, group, or community-based interventions. Middelweerd and colleagues [14] observed that the BCTs most frequently used in apps were self-regulation techniques (eg, goal setting, self-monitoring, and feedback on performance); furthermore, Direito et al [13] reported similar results.

To design tailored and targeted app-based interventions, insight into the preferences of the target population for certain BCTs applied in physical activity apps is of importance. Dennison et al [27] conducted focus group discussions with young adults and found that mobile phone features to track behavior, set goals, review progress, and receive feedback were positively evaluated. Moreover, Middelweerd et al [28] found that students prefer apps that motivate them and provide tailored feedback to achieve their personal exercise goals. Rabin et al [29] combined a quantitative survey with qualitative interviews among adults and found that adults preferred automatic tracking of physical activity and receiving feedback on exercise achievements. However, these three studies were mainly qualitative and conducted with small samples. Ehlers and Huberty [30] took a more quantitative approach and also identified self-regulation techniques as valued features, but in a sample of middle-aged women; thus, quantitative information on BCT ratings in young adults is lacking.

Thus far, studies have mainly focused on general preferences for BCTs. Because BCTs are targeting determinants of behavior, it is interesting to examine whether preferences for specific BCTs are associated with these determinants, such as self-efficacy. Furthermore, Verkooijen and De Bruijn reported [31] that the association between social comparison and physical activity is partly mediated by exercise self-identity in young adult women. Because social comparison is a BCT, the preference for this specific BCT and for other techniques might be associated with exercise self-identity. Moreover, Middelweerd et al [28] reported differences in preferences for goal setting and coaching features among participants meeting physical activity guidelines and those who did not meet guidelines. These results suggest that these preferences are associated with levels of physical activity. Lastly, personality traits are associated with physical activity [32,33], and this relationship may be mediated by behavioral determinants proposed in social cognitive models (eg, self-efficacy). As BCTs are techniques aimed at effectively changing behavioral determinants and subsequent behavior, it may be that personality influences the effectiveness of BCTs, probably due to differences in preferences and use of BCTs. Therefore, we explored the hypothesis that preferences for specific BCTs are associated with personality traits.

The aim of this paper is to explore young adults’ ratings of BCTs applied in a mobile phone physical activity app targeting self-efficacy and social support as important correlates of physical activity, and to explore whether these ratings are associated with personality characteristics (ie, personality, exercise self-efficacy, and exercise self-identity) and levels of physical activity.

##  Methods

### Design and Recruitment

**Figure 1:**
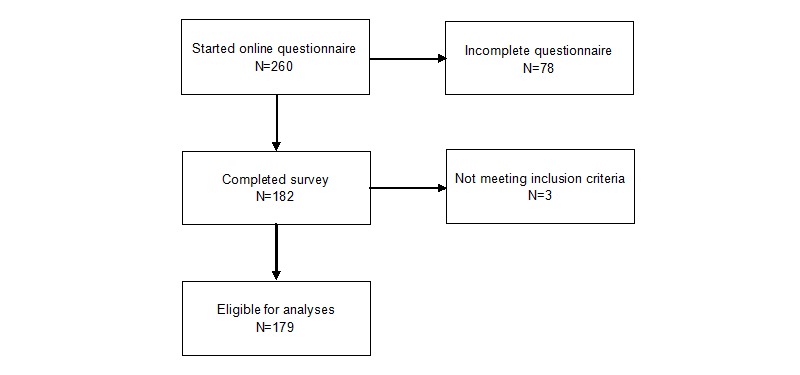
Data selection process for analyses.

Apparently healthy (in this case, narrowly defined as the absence of physical impairments) young adults, aged 18-30 years, were recruited to voluntarily complete an open online cross-sectional survey via email, Web-based advertising (eg, websites, social media, notification via the Vrije Universiteit Amsterdam [VU University] online communication platform), printed flyers with a link to the questionnaire (eg, universities, fitness centers, cafes), and personal approaches (eg, asking participants personally to complete the questionnaire) at both VU University and several secondary vocational education schools. Participants were informed that they would receive an incentive for their participation (ie, an activity tracker worth 90 Euros). Participants were eligible for inclusion if they met the age criteria (18-30 years) and if they did not report physical impairments that limited their physical activity (eg, a doctor’s order to not participate in any sports or physical activities); incomplete surveys (n=78) were excluded (Figure 1). A total of 260 individuals agreed to participate, but only 182 completed the survey in April 2014 (a completion rate of 70%). Data for 3 participants who had physical impairments were removed as this most likely influenced their amount of physical activity, leaving data for 179 eligible participants for analyses. The Medical Ethical Committee of VU University Medical Center approved the study. All participants gave informed consent. Seventy-eight participants did not complete the questionnaire, probably due to time constraints or technical issues.

### Procedures

The questionnaire was primarily based on existing validated instruments [34-37]. When no Dutch version of a questionnaire existed, it was translated into Dutch and back-translated into English by different translators to ensure correct interpretation of questions. The survey was pilot tested among 10 master’s students at VU University who met the inclusion criteria of the study. The online survey was administered through Survey Monkey [38] and data were downloaded to SPSS 20.0 (IBM) and filtered for survey completion and eligibility criteria, according to the protocol for online questionnaires (Checklist for Reporting Results of Internet E-Surveys, CHERRIES) [39]. Personal data were deleted to prevent unauthorized access and to ensure privacy.

### Measures

The 108-item questionnaire assessed demographics, personality traits [34], exercise self-efficacy [36], social support for physical activity [40], exercise self-identity [41-43], and physical activity levels [37]. Questions were included to indicate participants’ ratings of BCTs applied in a mobile phone physical activity app according to the 93-item taxonomy [22].

#### Behavior Change Techniques

Potentially effective BCTs to enhance self-efficacy and social support for physical activity were selected from the BCT taxonomy of Michie et al [22] and recent literature on potential effective BCTs [23-26,44-46].

First, participants were asked to indicate their general preference for functionalities of an app: a personal coach, self-monitoring of physical activity, both, or neither. Second, ratings of specific BCTs were measured on a 5-point Likert scale (Table 1), for which stronger agreement meant a more positive rating of the BCT. Exploratory factor analysis—principal components analysis with orthogonal rotation—confirmed that the 16 BCTs could be grouped into 3 categories: “goal setting and goal reviewing*”* (goal setting for behavior, problem solving, goal setting for the outcome of behavior, action planning, reviewing behavior goals, discrepancies between current behaviors and goals, reviewing outcomes of goals, and graded tasks, with factor loadings ranging from .510 to .786), “feedback and self-monitoring*”* (feedback on behavior and the outcome of behavior, self-monitoring of behavior, and the outcome of behavior, with factor loadings ranging from .635 to .811), and “social support and social comparison*”* (unspecified practical and emotional social support and social comparison, with factor loadings ranging from .508 to .921). The Cronbach alphas of the newly created scales showed good internal consistency (.86, .81, and .83 for “goal setting and goal reviewing,” “feedback and self-monitoring,” and “social support and social comparison,” respectively). Furthermore, Harman’s single-factor test showed that a single factor did not account for the majority of the covariance, indicating that it was not necessary to adjust for bias due to common method variance. Multimedia Appendix 1 shows details of the factor analysis and Harman’s single-factor test. Because of a skewed distribution, the variables “goal setting and goal reviewing” and “feedback and self-monitoring” were dichotomized at the second tertile.

**Table 1:** Selected behavior change techniques (BCTs) included in the online survey.

Selected BCT^a^	Question included in the survey
Goal setting (behavior)	It is important to me that I can set short-term goals in a PA app
Problem solving	It is important to me that I can solve a problem that holds me back from exercising in a PA app
Goal setting (outcome)	It is important to me that I can set long-term goals in a PA app
Action planning	It is important to me that I can plan my exercise activities in a PA app
Reviewing behavior goal(s)	It is important to me that I have an overview of my exercise goals to improve my PA in the short-term and can review my progress in a PA app
Discrepancies between current behaviors and goal(s)	It is important to me that I can see the difference between my current exercise behavior and my goals in a PA app
Reviewing outcome goal(s)	It is important to me that I have an overview of my long-term PA goal and can review my long-term goal progress in a PA app
Graded tasks	It is important to me that I can start with easy tasks and gradually make the exercise tasks more difficult in a PA app
Feedback on behavior	It is important to me that I get feedback on my level of PA in a PA app
Self-monitoring of behavior	It is important to me that I can monitor my exercise activities in a PA app
Self-monitoring of the outcome(s) of behavior	It is important to me that I can monitor my long-term results in a PA app
Feedback on the outcome(s) of behavior	It is important to me that I get feedback on my long-term results in a PA app
Social support (unspecified)	It is important to me that I can receive advice or support from friends, family, or colleagues in a PA app to exercise more
Social support (practical)	It is important to me that I can receive practical advice from friends, family, or colleagues in a PA app to exercise more
Social support (emotional)	It is important to me that I can be encouraged by friends, family, or colleagues in a PA app to exercise more
Social comparison	It is important to me that I can compare my exercise activities with that of others in a PA app

^a^Behavior change techniques (BCTs) based on the 93-taxonomy of Michie et al [22].

#### Personality

Personality traits were measured with the Dutch version of the 10-item short form of the Big Five Inventory (BFI) and the Ten-Item Personality Inventory (TIPI). The original TIPI [47] was translated from the English language, back-translated to confirm the translation, and validated among Dutch university students by Hofmans et al [34]. We measured the five tendencies of personality traits: extraversion (E), agreeableness (A), conscientiousness (C), neuroticism (N), and openness (O) [35] using a 7-point Likert scale (1= totally disagree, 7= totally agree) [34,35]. Each TIPI subscale includes 2 items representing opposite poles of each Big Five personality trait and each has a subscale score ranging from 1 to 7 [34].

#### Exercise Self-Efficacy

Exercise self-efficacy was measured with 12 questions on a 5-point Likert scale (1=I know I cannot, 5=I know I can) based on the Self-efficacy for Exercise Scale [36]. Test-retest reliability for this scale was reported as .68 [36]. Moreover, this study also showed good internal consistency (Cronbach alpha of .90).

#### Exercise Self-Identity

Exercise self-identity was measured with 4 questions on a 7-point Likert scale (1=totally disagree, 7=totally agree): “Engaging in sufficient exercise is something that fits the way I want to live,” “Engaging in sufficient exercise is something that fits who I am,” “I see myself as someone who engages in sufficient exercise,” and “I am a typical person who engages in sufficient exercise” [34,42,43]. A sum score of these constructs was constructed (ie, range 4-28) and showed good internal consistency (Cronbach alpha of .88).

#### Physical Activity

Physical activity was assessed with the SQUASH (ie, Short QUestionnaire to ASsess Health-enhancing physical activity), a validated Dutch questionnaire that measures different types and intensity of physical activity [37]. A test-retest analysis (5-week period) showed reproducibility of .58 (95% CI 0.36-0.74), with better reproducibility for high-intensity activities, such as active commuting and leisure time sports, than low-intensity activities, indicating that the SQUASH is a fairly reliable measure [37]. Moreover, the validity of the SQUASH was fair to good, as indicated by a correlation of .45 (95% CI 0.17-0.66) between the SQUASH and activity counts derived from activity monitors [38]. Physical activity at work or school was assessed in hours/day instead of hours/week and subsequently transformed to scores per week. The scores that were used from the SQUASH questionnaire were total physical activity (min/week) and meeting the Dutch physical activity recommendation (yes or no).

#### Mobile Phone and App Use

Items were included to measure mobile phone use (“Do you use a mobile phone?” [yes or no]), past mobile phone app use (“Did you use mobile phone apps in the past?” [yes or no]), current mobile phone app use (“Do you use mobile phone apps at the moment?” [yes or no]), and physical activity app use (“Do you use mobile phone apps focused on sports and exercise?” [yes, often; yes, sometimes; yes, seldom; or no]).

#### Demographics

Age, gender, nationality (ie, Dutch or non-Dutch), current level of education (ie, secondary vocational education, higher education, university, other), current residence, study city, living situation (ie, own place or with parents/family), ability to exercise (ie, yes, yes despite physical activity impairments, no), height (m), and weight (kg) were collected.

### Statistical Analyses

Multiple binary logistic regression analyses were used to estimate the association between the different personality characteristics (personality traits, exercise self-efficacy, exercise self-identity, and meeting the Dutch physical activity guidelines) and positive ratings (no, yes) of BCTs addressing “goal setting and goal reviewing” and “feedback and self-monitoring.” Multiple linear regression analyses were used to explore the association between personality characteristics and the preference for the BCTs addressing “social support and social comparison.” Potential effect moderation was evaluated for meeting the Dutch physical activity guidelines. The models were evaluated for potential confounds of total physical activity (min/week) and body mass index (BMI, kg/m^2^). For all associations, *P*≤.05 indicated a statistically significant association, except for the interaction terms, which were considered significant at *P*≤.10.

## Results

### Participant Characteristics

**Figure 2:**
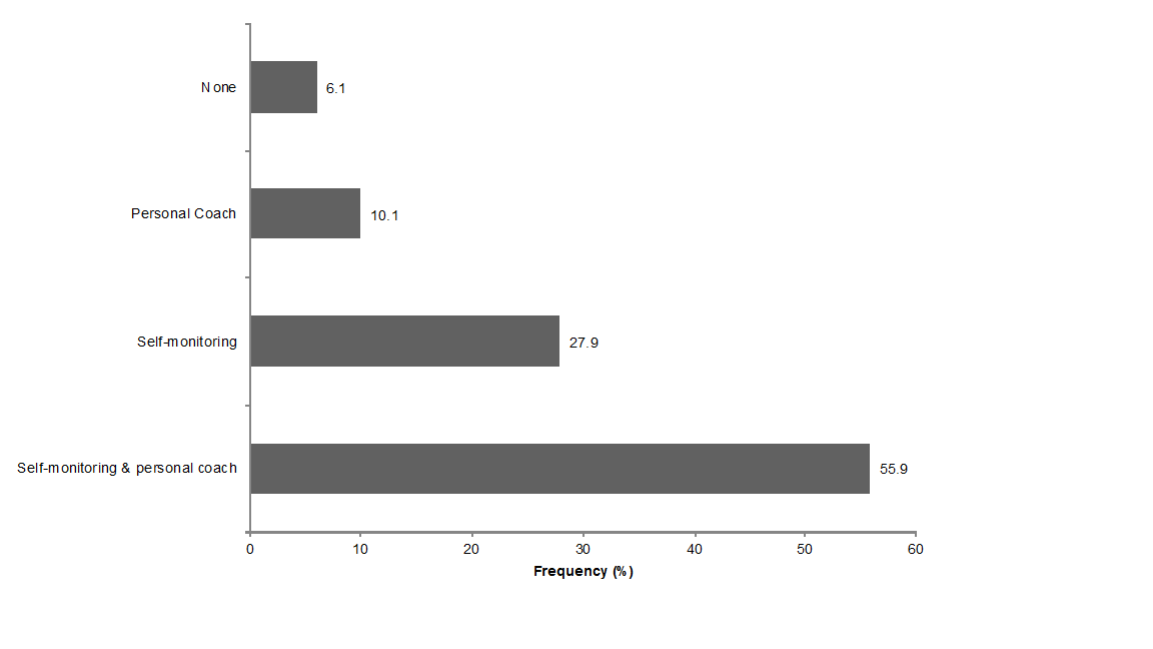
Physical activity app functionalities desired by participants.

Sample characteristics and mean scores of the independent and dependent variables for the total study population are presented in Table 2. Overall, most participants were female, highly educated, and physically active. Table 3 describes preferences for BCTs in physical activity apps, and Figure 2 describes the physical activity app functionalities desired by participants.

**Table 2:** Characteristics of participants.

Demographic characteristics		N=179
Gender, male, n (%)		33 (18.4%)
Age (years), mean (SD)		24.33 (±2.76)
Body mass index (kg/m^2^), mean (SD)		22.05 (±2.62)
Dutch nationality, n (%)		152 (84.9%)
**Current level of education, n (%)**		
	Secondary vocational education	5 (2.8%)
	Higher education	33 (18.4%)
	University	136 (76.0%)
	Other	5 (2.8%)
**Student, n (%)**		118 (65.9%)
	Living situation, on their own, n (%)	150 (83.8%)
	Mobile phone use, yes, n (%)	173 (96.6%)
	Past mobile phone app use, yes, n (%)	171 (95.5%)
	Current mobile phone app use, yes, n (%)	168 (93.9%)
**Physical activity app use, n (%)**		
	Yes, often	35 (19.6%)
	Yes, sometimes	56 (31.3%)
	Yes, seldom	25 (14.0%)
	No	63 (35.2%)
**Preference for BCTs** ^a^		
	Goal setting, goal reviewing (range 8-40), mean (SD)	31.12 (±6.18)
	Feedback, self-monitoring (range 4-20), mean (SD)	16.60 (±2.75)
	Social support, social comparison (range 4-20), mean (SD)	10.65 (±3.95)
Meet the Dutch physical activity recommendation of at least 30 minutes of moderate physical activity 5 days/week, n (%)		144 (80.4%)
**Physical activity hours/week, mean (SD)**		
	Total	48.03 (±21.57)
	Moderate to vigorous	16.60 (±17.64)
Exercise self-efficacy (range 12-60), mean (SD)		44.74 (±8.79)
Exercise self-identity (range 4-28), mean (SD)		21.64 (±5.00)
**Personality (range 1-7), mean (SD)**		
	Extraversion (E)	4.74 (±1.47)
	Agreeableness (A)	5.49 (±0.84)
	Conscientiousness (C)	4.86 (±1.40)
	Neuroticism (N)	3.05 (±1.24)
	Openness (O)	4.88 (±1.24)

^a^Behavior Change Technique.

**Table 3:** Mean preferences for behavior change techniques (BCTs) in a physical activity app^a^.

BCT		Mean (±SD)
**Goal setting and goal reviewing**		
	Goal setting for behavior	3.84 (±1.13)
	Problem solving	3.46 (±1.31)
	Goal setting for the outcome of behavior	4.18 (±0.93)
	Action planning	3.55 (±1.20)
	Review of behavior goals	3.85 (±1.01)
	Discrepancy between current behavior/goal	4.12 (±1.02)
	Review of the outcome of behavior goals	4.04 (±1.01)
	Graded tasks	4.07 (±0.98)
**Feedback and self-monitoring**		
	Feedback on behavior	3.93 (±0.95)
	Self-monitoring of behavior	4.41 (±0.75)
	Self-monitoring of the outcome of behavior	4.22 (±0.83)
	Feedback on the outcome of behavior	4.03 (±0.90)
**Social support and social comparison**		
	Social support unspecified	2.37 (±1.11)
	Social support practical	2.52 (±1.18)
	Social support emotional	2.63 (±1.30)
	Social comparison	3.13 (±1.26)

^a^Rated on a scale from 1 (strongly disagree) to 5 (strongly agree).

### Associations With Ratings of BCTs Addressing Goal Setting and Goal Reviewing

Table 4 shows that few personality characteristics were significantly associated with high ratings of the BCTs addressing goal setting and goal reviewing. Meeting the Dutch physical activity guidelines did not significantly moderate the association. “Agreeableness*”* was significantly positively associated with high ratings of the BCTs addressing goal setting and goal reviewing (OR 1.60, 95% CI 1.06-2.41), indicating that respondents who scored 1 point higher on agreeableness (range 1-7) were 1.60 times more likely to rate this BCT category as important.

**Table 4:** Association between personality traits and a high preference for behavior change techniques addressing goal setting and goal reviewing^a^.

Characteristic		Unadjusted odds ratio (95% CI)	*P*	Nagelkerke *R* ^2^	Adjusted odds ratio (95% CI)	*P*	Nagelkerke *R* ^2^
**Personality traits**							
	Extraversion (E)	1.08 (0.86-1.36)	.519	.066	1.06 (0.84-1.34)^b^	.636	.066
	Agreeableness (A)	1.61 (1.07-2.43)	.022		1.60 (1.06-2.41)^b^	.026	
	Conscientiousness (C)	0.86 (0.68-1.08)	.183		0.86 (0.68-1.10)^b^	.237	
	Neuroticism (N)	1.07 (0.82-1.39)	.626		1.04 (0.80-1.37)^b^	.755	
	Openness (O)	1.09 (0.84-1.42)	.513		1.09 (0.84-1.43)^b^	.514	
Exercise self-efficacy		1.00 (0.96-1.03)	.827	<.001	1.00 (0.96-1.03)^b^	.823	.007
Exercise self-identity		0.99 (0.93-1.05)	.649	.002	0.99 (0.93-1.06)^b^	.773	.007
Meeting the Dutch physical activity guidelines		0.83 (0.39-1.78)	.636	.002	0.88 (0.40-1.92)^c^	.747	.006

^a^Categorized on the 2nd tertile (sum score of 8 questions on a 5-point Likert scale; 8-33 considered low and 34-40 considered high preference).

^b^Adjusted for total physical activity (min/week) and body mass index (kg/m^2^)

^c^Adjusted for body mass index (kg/m^2^)

### Associations With Ratings of BCTs Addressing Feedback and Self-Monitoring

Table 5 shows that no significant associations were found between BCT feedback and self-monitoring and exercise self-identity and meeting Dutch physical activity guidelines. A significant negative association was found with “neuroticism*”* (OR 0.76, 95% CI 0.58-1.00) and a significant positive association was found with exercise self-efficacy (OR 1.06, 95% CI 1.02-1.11), indicating that respondents who scored 1 point higher on neuroticism (range 1-7) were 1.32 times less likely to rate this BCT category as important, and respondents who scored 1 point higher on the 60-point exercise self-efficacy scale were 1.06 times more likely to rate “feedback and self-monitoring” as important BCTs. Meeting the Dutch physical activity guidelines did not moderate the associations.

**Table 5:** Association between personality traits and a high preference for behavior change techniques addressing feedback and self-monitoring^a^.

Characteristic		Unadjusted Odds Ratio (95% CI)	*P*	Nagelkerke *R* ^2^	Adjusted Odds Ratio (95% CI)	*P*	Nagelkerke *R* ^2^
**Personality traits**							
	Extraversion (E)	1.10 (0.88-1.39)	.407	.061	1.07 (0.84-1.35)^b^	.585	.073
	Agreeableness (A)	1.10 (0.74-1.61)	.645		1.08 (0.73-1.60)^b^	.705	
	Conscientiousness (C)	0.86 (0.69-1.08)	.188		0.84 (0.70-1.06)^b^	.141	
	Neuroticism (N)	0.80 (0.61-1.04)	.101		0.76 (0.58-1.00)^b^	.054	
	Openness (O)	1.12 (0.86-1.46)	.382		1.12 (0.87-1.46)^b^	.414	
Exercise self-efficacy		1.06 (1.02-1.10)	.003	.071	1.06 (1.02-1.11)^c^	.003	.088
Exercise self-identity		1.04 (0.98-1.10)	.241	.010	1.05 (0.98-1.12)^c^	.157	.029
Meeting the Dutch PA guidelines		0.99 (0.46-2.12)	.983	<.001	1.07 (0.49-2.32)^d^	.867	.007

^a^Categorized on the 2^nd^ tertile (sum score of 4 questions on a 5-point Likert scale, 4-17 low and 18-20 high preference).

^b^Adjusted for total physical activity (min/week)

^c^Adjusted for total physical activity (min/week) and body mass index (kg/m^2^)

^d^Adjusted for body mass index (kg/m^2^)

### Associations With Ratings of BCTs Addressing Social Support and Social Comparison

Data obtained from the linear regression analysis between personal characteristics and ratings of BCTs addressing social support and social comparison (range 4-20) are presented in Table 6. No significant associations were found and meeting the Dutch physical activity guidelines was not a significant moderator.

**Table 6:** Association between personality characteristics and preference for behavior change techniques addressing social support and social comparison.

Characteristic		Standardized B (95% CI)	*P*	Nagelkerke *R* ^2^	Adjusted standardized B (95% CI)	*P*	Nagelkerke *R* ^2^
**Personality traits**							
	Extraversion (E)	0.08 (-0.08 to 0.23)	.329	.046	0.07 (-0.10 to 0.23)^a^	.430	.049
	Agreeableness (A)	0.10 (-0.06 to 0.25)	.217		0.09 (-0.06 to 0.24)^a^	.254	
	Conscientiousness (C)	-0.13 (-0.28 to 0.02)	.087		-0.14 (-0.30 to 0.03)^a^	.103	
	Neuroticism (N)	-0.01 (-0.16 to 0.15)	.907		-0.02 (-0.18 to 0.14)^a^	.821	
	Openness (O)	0.09 (-0.07 to 0.24)	.280		0.08 (-0.07 to 0.24)^a^	.299	
Exercise self-efficacy		0.08 (-0.07 to 0.23)	.308	.006	0.09 (-0.06 to 0.24)^a^	.266	.015
Exercise self-identity		-0.04 (-0.19 to 0.11)	.605	.002	-0.03 (-0.18 to 0.12)^b^	.696	.005
Meeting the Dutch physical activity guidelines		0.08 (-0.07 to 0.22)	.310	.006	0.09 (-0.06 to 0.24)^b^	.229	.013

^a^Adjusted for total physical activity (min/week) and body mass index (kg/m^2^).

^b^Adjusted for body mass index (kg/m^2^).

##  Discussion

### Principal Findings

This study examined young adults’ ratings of BCTs applied in a mobile phone physical activity app aimed at improving self-efficacy and social support for physical activity. Furthermore, a number of possible correlates of such ratings were explored. It should be noted that participants were asked to rate BCTs based on their experiences and wishes or requirements of a hypothetical mobile phone physical activity app and not of an existing app. In this study, the ratings of all BCTs addressing self-efficacy were relatively high, but the BCTs addressing social support were not. Respondents who scored higher on agreeableness were more likely to rate BCTs addressing “goal setting and goal reviewing” positively. Furthermore, respondents who scored higher on neuroticism were less likely to rate BCTs addressing “feedback and self-monitoring” positively, while higher scores on self-efficacy were associated with more positive ratings of “feedback and self-monitoring.”

### Ratings of Behavior Change Techniques

This study’s finding that BCTs addressing “goal setting and goal reviewing” and “feedback and self-monitoring” were positively rated is in line with previous research, in which app features for (automatic) tracking of behavior, setting goals, monitoring behavior, and receiving feedback were evaluated positively in different studies [27,29]. In this study, the most preferred BCTs by young adults were “goal setting on the outcome of behavior,” “self-monitoring of behavior,” and “self-monitoring of the outcome of behavior.” The study of Ehlers and Huberty [30] supports this finding, by indicating that self-regulation techniques (eg, tracking physical activity, goal setting, and receiving feedback) are valuable features of health behavior apps. Middelweerd et al [28] pointed out that participants, overall, preferred a combination of a (virtual) coach with goal setting and that participants would like to receive personal feedback. This study provides further support for positive ratings of BCTs, including goal setting and receiving personal feedback. Earlier findings that social support features were less appreciated among middle-aged women were replicated for young adults, based on the lower preference for social support techniques in apps found in this study [30]. It may be that the high physical activity and exercise self-efficacy levels of the participants in this study caused them to perceive social support as unnecessary. By contrast, about half of the participants preferred a personal coach, which could be seen as another form of social support. Additional analyses (not shown) indicated that those preferring a personal coach did not significantly differ with respect to their preferences for social support from those who did not prefer a personal coach.

### Personal Characteristics and Ratings of Behavior Change Techniques

The association between personality traits and BCT categories has not been previously reported or investigated. This study found a significant positive association for agreeableness and BCTs addressing “goal setting and reviewing.” Agreeableness is characterized by having the tendency to be kind, cooperative, and trustworthy [37]; thus, participants who were categorized as being cooperative and trustworthy were more likely to be open to app features like goal setting and goal reviewing. This study found an inverse association between neuroticism and BCTs addressing “feedback and self-monitoring.” Neuroticism is characterized by negative affect and emotional instability [48]. Rhodes and Smith [32] noted that avoidance of physical activity or cancelling physical activity plans may be a logical extension of this personality trait, which can also make them less likely to use app features like goal setting, goal reviewing, receiving feedback, and self-monitoring of physical activity behavior. These preliminary results indicate that personality traits could be considered when designing app-based interventions. Previous research showed that tailoring advertising messages to respondents’ personality traits increased their motivation to use a product. Thus, tailoring BCTs to participants may increase the effectiveness of an app-based intervention. However, future research is needed to examine how BCTs should be tailored.

Furthermore, this study found an association between exercise self-efficacy and BCTs addressing “feedback and self-monitoring.” It may be that when a person perceives that one can successfully engage in physical activity, monitoring physical activity and receiving feedback on physical activities affirm and ratify a positive feeling toward physical activity behavior. Moreover, when participants’ exercise self-efficacy is low, monitoring their physical activity behavior may elicit unpleasant feelings and, therefore, generate less appreciation for self-monitoring app features. This suggests that app developers should consider designing app-based interventions tailored to personality and exercise self-efficacy.

Although this study did not find any associations between participants meeting the Dutch physical activity guidelines and ratings of potential effective BCTs, Middelweerd and colleagues [28] found some differences in ratings of app features between young adults who met and who did not meet these guidelines. For example, the latter preferred a goal setting feature and a personal coach, whereas participants meeting the physical activity guidelines reported goal setting as unnecessary and preferred highly detailed training information [28]. Furthermore, differences may also exist between men and women, as men appear to have a preference for team-based, competitive activities, while women do not [49]. The lack of association with participants’ physical activity level could also be explained in terms of a high overall level of physical activity in this study, as more than 80% of participants met the guidelines; thus, this study may not have had sufficient power to detect differences in preferences for BCTs between those meeting and not meeting the Dutch guidelines.

### Strengths and Limitations

This is the first study to explore associations between personality characteristics and ratings of BCTs applied in mobile phone physical activity apps. Given the current lack of adequate physical activity and preference for apps to support and monitor physical activity, scientific evidence to inform app-based interventions is needed.

Several limitations need to be considered in the interpretation of the findings of this study. The first important limitation is the cross-sectional design, so no causal inferences can be made. Second, the sample was rather homogeneous in gender, physical activity level, and education level; therefore, the results cannot be generalized to more heterogeneous samples. The fact that participants were highly active and had high levels of self-efficacy may have influenced their preferences for certain BCTs. Reasons for the homogenous sample in this study could be that the participants comprised a convenience sample, and the study’s topic (physical activity and apps) and the activity tracker incentive may have attracted more physically active participants. An additional incentive to participate in the study was a prize draw in which one activity tracker could be won. Such an incentive was probably more attractive to participants who already were physically active and who may already have had a preference for tracking their behavior. This may have influenced their preferences for certain BCTs. Consequently, the high levels of preferences for self-monitoring BCTs might be somewhat biased. The time and effort needed to complete the survey may have led to lower participation of less educated participants [50], despite efforts to actively recruit participants at secondary vocational education schools to reach participants with lower education. However, it is well-known that those with lower education and who are physically inactive are hard to reach. Furthermore, men are less likely to participate in lifestyle-related research [51-55]. The fact that our sample was mainly female, with higher education, and more likely to be physically active than the general population of Dutch young adults may have biased the results, and the BCTs identified may not be reflective of the population at large. Furthermore, this study examined a relatively large number of correlations, but only 3 associations were found to be significant, resulting in increased risk of Type 1 error. These associations should, thus, be interpreted with caution and regarded as exploratory findings. Finally, the participants were asked to rate BCTs based on a one-sentence description, and not on actual experience. Consequently, for some participants, past experiences might have positively or negatively influenced their ratings, whereas for others the ratings remained hypothetical.

### Future Research

Future research should focus further on indicating potential differences in the ratings of BCTs between active and inactive participants and using more representative samples. Self-regulation BCTs are potentially effective and were highly appreciated among the young adults in this study. Therefore, these techniques may be considered by physical activity app developers, who should implement these BCTs correctly; the apps should be subsequently tested for their effectiveness in improving physical activity motivation, self-efficacy, and behavior [56]. Although the literature indicates social support as an important correlate of physical activity, young adults in this study did not appreciate this in a mobile phone app. This may indicate that social support should be provided through different or traditional interventions. In addition, this study suggests the value of studying in detail the tailoring of BCTs to participants’ personality characteristics.

Future research should examine the effectiveness of BCTs applied in apps. In previous research, it has been shown that the BCTs included in this study can effectively change behavior; however, because of lack of evaluation research, little is known about their effectiveness in apps [14]. Even if BCTs have been found to be effective in other intervention methods, effectiveness in app operationalization should be tested, as effectiveness of BCTs may be dependent on the actual intervention.

### Conclusion

To conclude, ratings of various self-regulation BCTs in a mobile phone app were high in a selected group of highly educated and physically active young adults. BCTs addressing social support were less appreciated. Differences in ratings of BCTs due to differences in personality and exercise self-efficacy between young adults should be taken into account.

## Appendix

Multimedia Appendix 1 Summary of exploratory factor analysis results for the behavior change techniques (N=179).

## Abbreviations

BCT: behavior change technique
BFI: big five inventory
TIPI: ten-item personality inventory
