# Supplementary material for: Dutch Young Adults Ratings of Behavior Change Techniques Applied in Mobile Phone Apps to Promote Physical Activity: A Cross-Sectional Survey
Source: JMIR Mhealth Uhealth. 2015 Nov 12;3(4):e103. doi: 10.2196/mhealth.4383 (PMC4704888; doi:10.2196/mhealth.4383)
Supplement: Multimedia Appendix 1 [file mhealth_v3i4e103_app1.pdf]

Multimedia Appendix 1

Summary of exploratory factor analysis results for the behavior change techniques (N=179).

	Rotated Factor Loadings[a]		
**Item**	Factor 1 (Goals setting and goal reviewing)	Factor 2 (Feedback and self-monitoring)	Factor 3 (Social support and social comparison)
It is important to me that I can set short-term goals in a PA app	**0.79**		
It is important to me that I can start with easy tasks and gradually make the exercise tasks more difficult in a PA app	**0.76**		
It is important to me that I have an overview of my exercise goals to improve my PA in the short-term and can review my progress in a PA app	**0.73**	0.35	
It is important to me that I can set long-term goals in a PA app	**0.71**	**0.43**	
It is important to me that I can solve a problem that holds me back from exercising in a PA app	**0.64**		
It is important to me that I have an overview of my long-term PA goal and can review my long-term goal progress in a PA app	**0.64**	**0.48**	
It is important to me that I can see the difference between my current exercise behavior and my goals in a PA app	**0.62**	**0.42**	
It is important to me that I can plan my exercise activities in a PA app	**0.51**		
It is important to me that I can monitor my long-term results in a PA app	0.38	**0.81**	
It is important to me that I get feedback on my long-term results in a PA app	**0.40**	**0.75**	
It is important to me that I can monitor my exercise activities in a PA app		**0.73**	
It is important to me that I get feedback on my level of PA in a PA app		**0.66**	
It is important to me that I can receive advice or support from friends, family, or colleagues in a PA app to exercise more			**0.92**
It is important to me that I can be encouraged by friends, family, or colleagues in a PA app to exercise more			**0.90**
It is important to me that I can receive practical advice from friends, family, or colleagues in a PA app to exercise more			**0.89**
It is important to me that I can compare my exercise activities with that of others in a PA app			**0.51**
**Eigenvalues**	**5.9**	**2.6**	**1.4**
**% of variance**	**34.4**	**14.9**	**5.4**
**α**	**0.86**	**0.81**	**0.83**

[a]Criterion level for factor loadings was 0.4 (marked bold).

Results of Harman’s single-factor test

Item	Factor 1 (All BCTs [a])
It is important to me that I can set short-term goals in a PA app	.664
It is important to me that I can solve a problem that holds me back from exercising in a PA app	.477
It is important to me that I can set long-term goals in a PA app	.792
It is important to me that I can plan my exercise activities in a PA app	.529
It is important to me that I have an overview of my exercise goals to improve my PA in the short-term and can review my progress in a PA app	.791
It is important to me that I can see the difference between my current exercise behavior and my goals in a PA app	.740
It is important to me that I have an overview of my long-term PA goal and can review my long-term goal progress in a PA app	.790
It is important to me that I can start with easy tasks and gradually make the exercise tasks more difficult in a PA app	.699
It is important to me that I get feedback on my level of PA in a PA app	.561
It is important to me that I can monitor my exercise activities in a PA app	.456
It is important to me that I can monitor my long-term results in a PA app	.795
It is important to me that I get feedback on my long-term results in a PA app	.762
It is important to me that I can receive advice or support from friends, family, or colleagues in a PA app to exercise more	.297
It is important to me that I can receive practical advice from friends, family, or colleagues in a PA app to exercise more	.335
It is important to me that I can be encouraged by friends, family, or colleagues in a PA app to exercise more	.316
It is important to me that I can compare my exercise activities with that of others in a PA app	.094
**% of variance**	**36.9**
**α**	**0.86**

[a] BCTs= Behavior change techniques
